# Supplementary material for: Measles Virus-Based Vaccine Expressing Membrane-Anchored Spike of SARS-CoV-2 Inducing Efficacious Systemic and Mucosal Humoral Immunity in Hamsters
Source: Viruses. 2024 Apr 3;16(4):559. doi: 10.3390/v16040559 (PMC11054861; doi:10.3390/v16040559)
Supplement: Supplementary file 1 [file viruses-16-00559-s001.zip › viruses-2929163-supplementary.pdf]

viruses

MDPI

Supplementary Figures

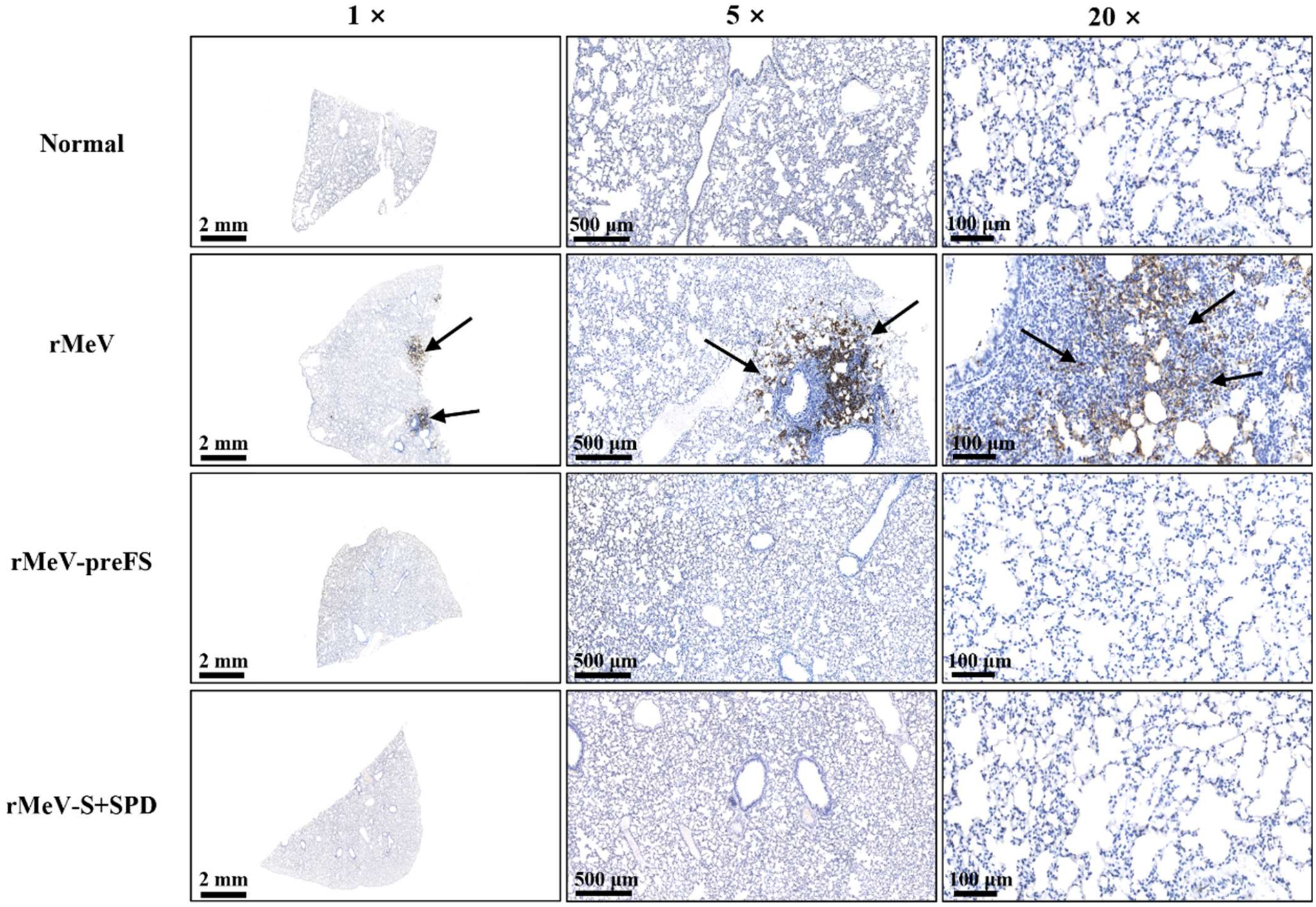

Figure S1. Omicron BA.2 viral protein expression in lungs of hamsters. IHC staining of SARS-CoV-2 N protein in the lung tissues of hamsters were shown. Micrographs of 1 ×, 5 ×, and 10 × magnifications of a representative lung section from each group are shown. Omicron BA.2 viral protein expression was indicated by arrows. Scale bars are indicated at the left corner of each image.
